# Supplementary material for: Compulsivity Reveals a Novel Dissociation between Action and Confidence
Source: Neuron. 2017 Oct 11;96(2):348–354.e4. doi: 10.1016/j.neuron.2017.09.006 (PMC5643443; doi:10.1016/j.neuron.2017.09.006)
Supplement: Document S1. Figures S1–S4 and Tables S1–S4 [file mmc1.pdf]

Neuron, Volume *96*

Supplemental Information

Compulsivity Reveals a Novel Dissociation between Action and Confidence

Matilde M. Vaghi, Fabrice Luyckx, Akeem Sule, Naomi A. Fineberg, Trevor W. Robbins, and Benedetto De Martino

Supplementary Material

Table S1. Demographic and Clinical Characteristics of the Participants Included in the Study. Related to STAR Methods.

	Controls	OCD Patients		
Gender (M:F)	13:12	13:11	$\chi^2_1 = 0.023$	$p = 0.879$
Age	40.68 (10.19)	41.33 (12.32)	$t_{47} = -0.203$	$p = 0.840$
Verbal IQ	117.92 (3.97)	116.32 (2.99)	$t_{46} = 1.568$	$p = 0.124$
MADRS	1.36 (2.02)	9.92 (4.36)	$t_{47} = -8.870$	$p < 0.001$
OCI-R	7.64 (6.37)	29.25 (11.87)	$t_{47} = -7.984$	$p < 0.001$
STAI-State	28.90 (7.06)	43.50 (10.72)	$t_{47} = -5.496$	$p < 0.001$
STAI-Trait	35.00 (7.01)	56.58 (7.91)	$t_{47} = -10.120$	$p < 0.001$
Y-BOCS Total	-	22.75 (4.32)		
Y-BOCS Obsessions	-	10.79 (2.41)		
Y-BOCS Compulsions	-	11.96 (2.23)		

Mean values are reported and standard deviation in brackets.

Table S2. List of Quantiles for Error Magnitude and Values of Learning Rate for Each Quantile. Related to Figure 2B.

Quantile	Boundaries quantiles	CTL		OCD	
		Mean	(SEM)	Mean	(SEM)
**1**	[1-2)	0.000	(0.00)	0.088	(0.053)
**2**	[2-3)	0.010	(0.017)	0.281	(0.082)
**3**	[3-4)	0.038	(0.032)	0.357	(0.142)
**4**	[4-5)	0.052	(0.034)	0.334	(0.114)
**5**	[5-7)	0.075	(0.038)	0.342	(0.094)
**6**	[7-8)	0.110	(0.049)	0.376	(0.097)
**7**	[8-9)	0.106	(0.055)	0.334	(0.090)
**8**	[9-11)	0.112	(0.034)	0.352	(0.083)
**9**	[11-12)	0.121	(0.042)	0.366	(0.090)
**10**	[12-13)	0.154	(0.055)	0.370	(0.087)
**11**	[13-15)	0.184	(0.042)	0.420	(0.077)
**12**	[15-17)	0.225	(0.049)	0.455	(0.077)
**13**	[17-19)	0.288	(0.044)	0.506	(0.063)

**14**	[19-22)	0.402	(0.043)	0.588	(0.047)
**15**	[22-25)	0.484	(0.044)	0.689	(0.041)
**16**	[25-29)	0.521	(0.043)	0.731	(0.036)
**17**	[29-37)	0.632	(0.045)	0.826	(0.040)
**18**	[37-66)	0.764	(0.037)	0.917	(0.035)
**19**	[66-125)	0.917	(0.020)	0.929	(0.019)
**20**	[125-180)	0.883	(0.021)	0.927	(0.019)

For values presented in Figure 2B the distribution of the values of the spatial prediction error (i.e., $\hat{\delta}_t = X_t - b_t$, Equation 2) was divided in 20 quantiles. The table displays boundaries of each quantile, and associated mean (and SEM in brackets) of the learning rate ($\hat{\alpha}$) for controls and OCD patients.

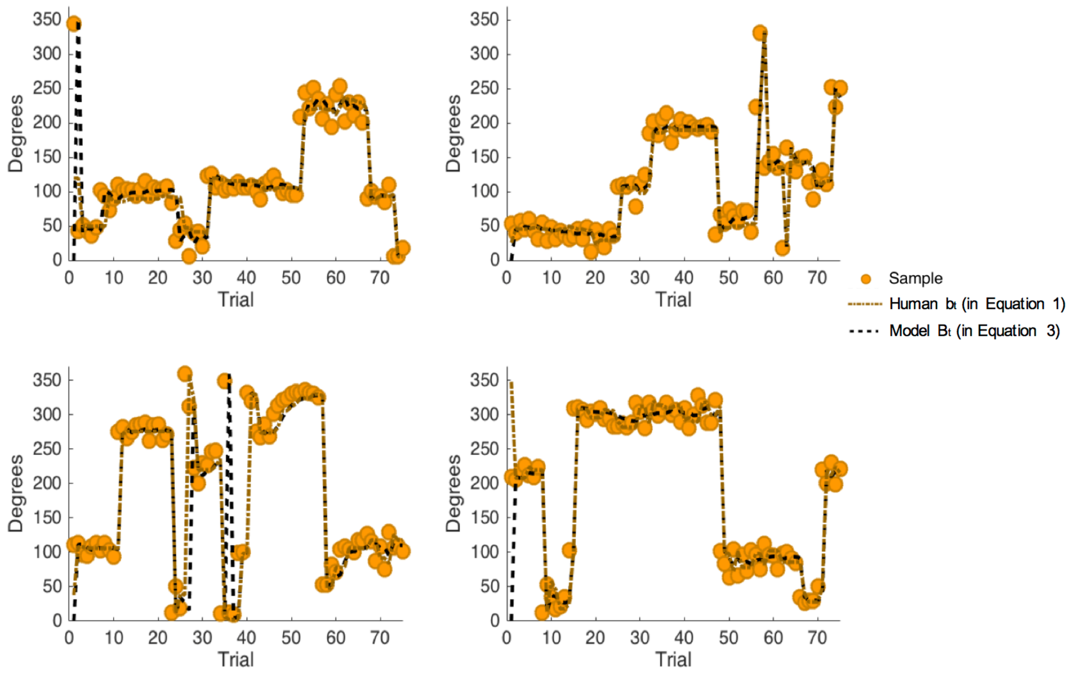

Figure S1. Comparison Between Human Participants and Model. Related to Figure 3B. Comparison was performed at the trial-by-trial level between subject's bucket position b_t computed in Equation 1 and model term B_t computed with Equation 3. Orange data points mark the location at which successive particles landed. The black dashed line marks the prediction of a quasi-optimal Bayesian model (i.e., B_t model belief update computed according to Equation 3, $B_{t+1} = B_t + \alpha_t \times \delta_t$). The brown dashed line marks the bucket's position (i.e., b_t in Equation 1 where participant positioned the bucket from one trial to the

next). Data are shown for one representative control subject. Each panel represents an experimental run of 75 trials.

Table S3. Summary of Parameters Obtained from the Regression Model for Action and Confidence[a]. Related to Figures 3C and 3F.

	Group	Beta	SEM	Within groups comparison			Between groups comparison		
					Stat	p	z	Rank sum	p
**Action**									
PE	CTL	0.264	0.077	t_{24}=	3.422	0.002	-2.370	506	0.018
	OCD	0.567	0.069	t_{23}=	8.197	<0.001			
CPP	CTL	0.565	0.056	t_{24}=	10.076	<0.001	2.530	752	0.011
	OCD	0.314	0.065	t_{23}=	4.856	<0.001			
MC	CTL	0.856	0.191	t_{24}=	4.479	<0.001	0	625	1
	OCD	0.632	0.130	t_{23}=	4.859	<0.001			
Hit/Missed	CTL	-0.742	0.053	t_{24}=	-13.978	<0.001	1.650	708	0.098
	OCD	-0.863	0.052	t_{23}=	-16.586	<0.001			
**Confidence**									
PE	CTL	-0.061	0.034	t_{24}=	-1.786	0.087	0.370	644	0.711
	OCD	-0.060	0.039	t_{23}=	-1.538	0.138			
CPP	CTL	-0.205	0.059	t_{24}=	-3.478	0.002	-1.170	566	0.242
	OCD	-0.107	0.049	t_{23}=	-2.157	0.042			
MC	CTL	-0.214	0.041	t_{24}=	-5.281	<0.001	-1.230	563	0.219
	OCD	-0.139	0.030	t_{23}=	-4.642	<0.001			
Hit/Missed	CTL	0.204	0.030	t_{24}=	6.854	<0.001	-0.330	608	0.741
	OCD	0.213	0.027	t_{23}=	7.832	<0.001			

[a] PE, prediction error; CPP, change-point probability; MC, model confidence.

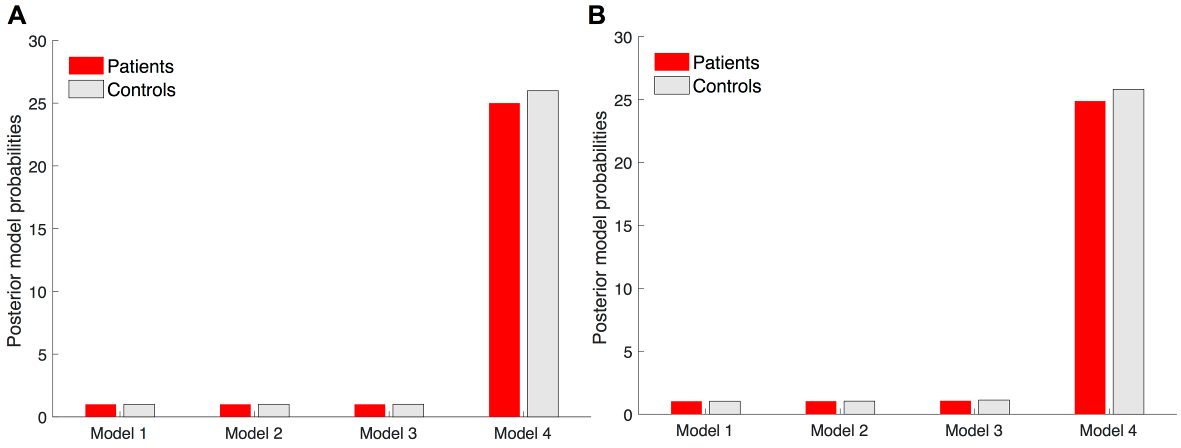

Figure S2. Bayesian Model Selection. Related to STAR Methods. (A) Posterior 'action regression' model probabilities after Bayesian model selection. The full and partial regression models predicting action were compared using the spm_BMS function (Stephan et al., 2009) from the SPM12 toolbox in Matlab (http://www.fil.ion.ucl.ac.uk/spm/software/spm12/). For both groups, the full regression model (Model 4, Table S4, Action) was deemed the most probable model. **(B)** Posterior 'confidence regression' model probabilities after Bayesian model selection. The best model to explain the data for both groups was again Model 4 (Table S4, Confidence), the full model with all four predictors included.

Table S4. Formulae for Models Represented in Figure S2 [a]. Related to STAR Methods.

Models	Formulae
**Action**	
**Model 1**	Action ~ $ß_0$ + $ß_1$[Prediction error] + ε
**Model 2**	Action ~ $ß_0$ + $ß_1$[Prediction error] + $ß_2$[CPP*PE] + ε
**Model 3**	Action ~ $ß_0$ + $ß_1$[Prediction error] + $ß_2$[CPP*PE]<br>+ $ß_3$[(1-CPP)*(1-MC)*PE] + ε
**Model 4**	Action ~ $ß_0$ + $ß_1$[Prediction error] + $ß_2$[CPP*PE]<br>+ $ß_3$[(1-CPP)*(1-MC)*PE] + $ß_4$[Hit*PE] + ε
**Confidence**	
**Model 1**	Confidence ~ $ß_0$ + $ß_1$[Prediction error] + ε
**Model 2**	Confidence ~ $ß_0$ + $ß_1$[Prediction error] + $ß_2$[CPP] + ε
**Model 3**	Confidence ~ $ß_0$ + $ß_1$[Prediction error] + $ß_2$[CPP] + $ß_3$[(1-CPP)*(1-MC)] + ε
**Model 4**	Confidence ~ $ß_0$ + $ß_1$[Prediction error] + $ß_2$[CPP] + $ß_3$[(1-CPP)*(1-MC)] + $ß_4$[Hit] + ε

[a]PE, prediction error; CPP, change-point probability; MC, model confidence.

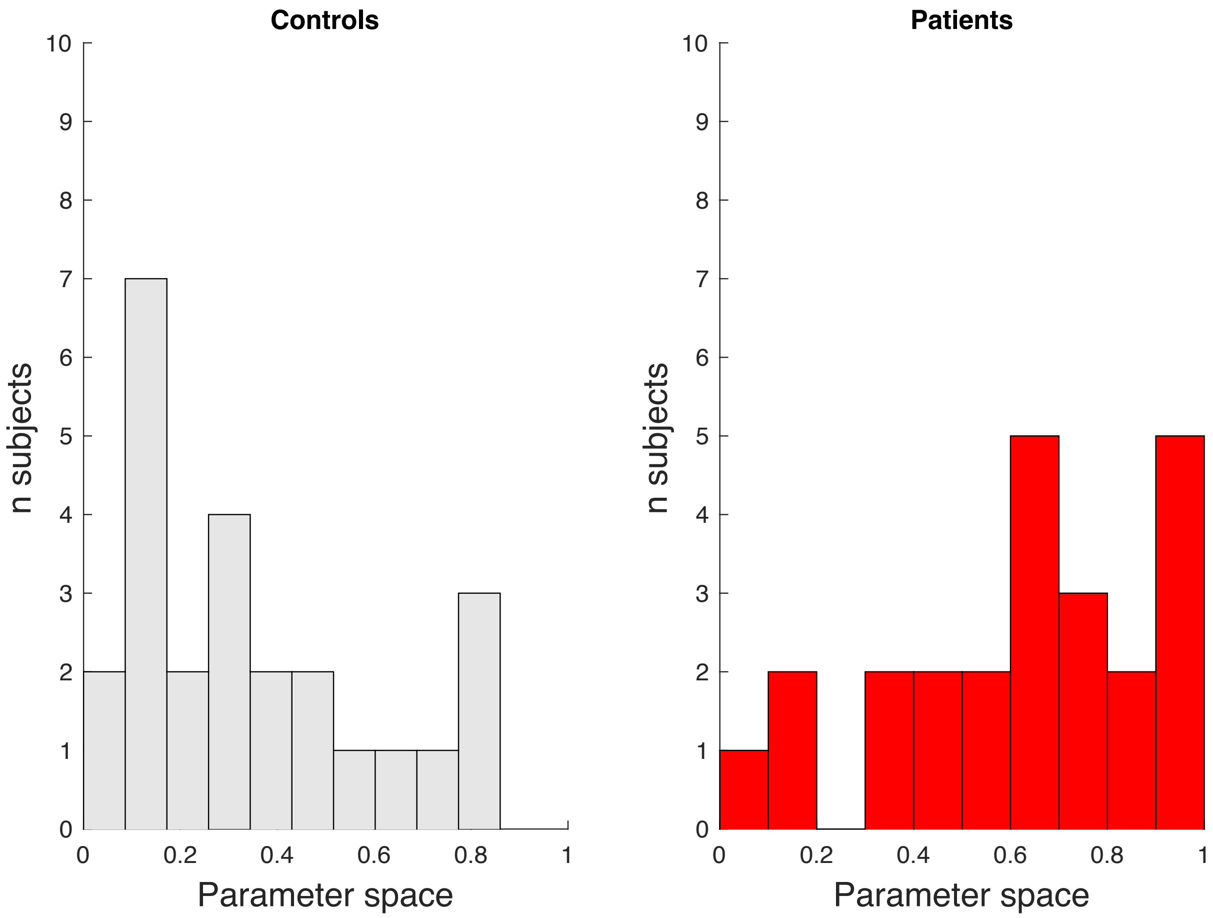

Figure S3. Distribution of Fitted Hazard Rates for Each Group. Related to STAR Methods. Model fits were obtained after an exhaustive search for possible values between 0 and 1. Best fitting parameters were determined by minimum least-squares fits between subject and model choices on each trial. The distribution of model fits showed a clear bimodal distribution, with overall higher hazard rate fits for patients (median = .62) compared to controls (median = .32, Wilcoxon rank-sum test, p = .007). However, these results are not surprising, as hazard rate is highly correlated with learning rate (see Nassar et al., 2012). Furthermore, we did not include these fits in our main analyses as those focused on subjects' behavior compared to a benchmark Bayesian model.

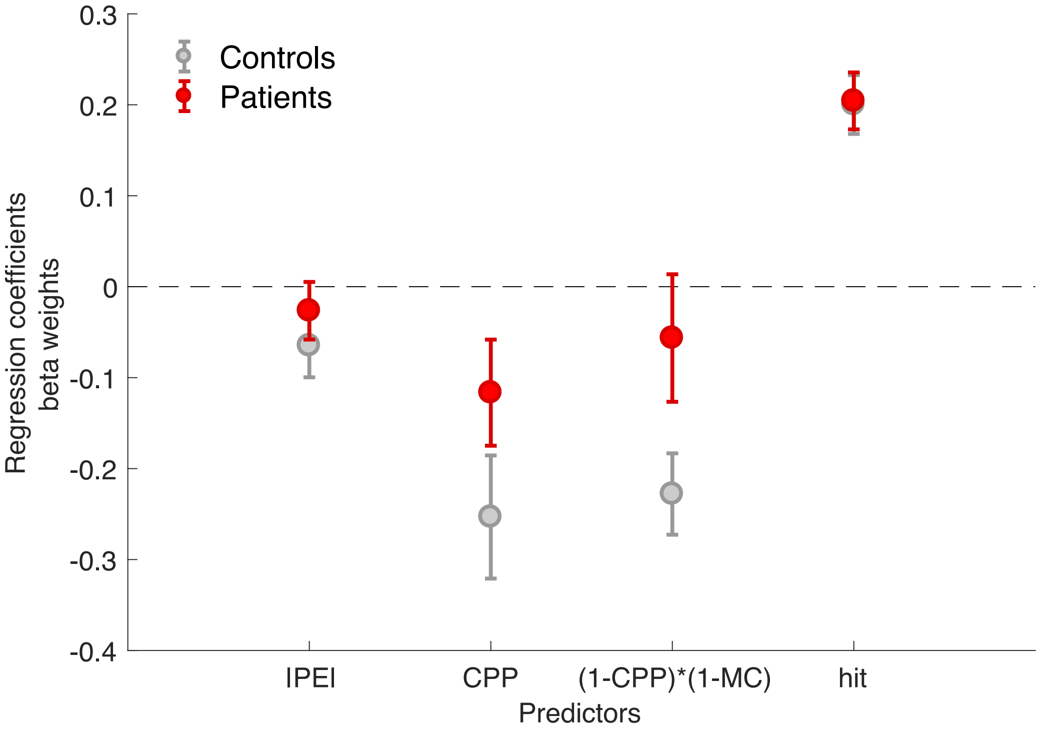

Figure S4. Regression on Confidence for Model with Fitted Hazard Rate. Related to STAR Methods. We ran an identical regression model as Figure 3F, with one change: model regressors CPP and (1-CPP)*(1-MC) were no longer derived from the benchmark Bayesian model, but from the model with fitted hazard rate for each participant. Results showed that model confidence no longer predicted patients' confidence (MC: OCD, -0.06±0.07, t_{19}=0.79, p=0.44), and this was significantly higher compared to controls (CTL, -0.23±0.04; paired two-sided t-test, p=0.04). Error bars represent SEM. Note that four participants from the patient group were excluded from the analysis, as their hazard rate was close to 1, in which case the two model predictors are perfectly negatively correlated and the regression model cannot find good fits. This analysis provides a different way of showing the dissociation of action and confidence in the OCD group: when hazard rate is fitted to participants' action (i.e. bucket update) OCD behavior is fitted by higher hazard rate, in other words OCD actions were reflecting an increased *perceived* volatility in the environment. However, OCD confidence rating did not reflect this increased *perceived* volatility (estimated using the fitted model) but were more aligned with the actual level of volatility in the environment. This analysis might provide another indication that patients did indeed understand the properties of the environment, but failed to act upon them.
