# Supplementary material for: Compulsivity Reveals a Novel Dissociation between Action and Confidence
Source: Neuron. 2017 Oct 11;96(2):348–354.e4. doi: 10.1016/j.neuron.2017.09.006 (PMC5643443; doi:10.1016/j.neuron.2017.09.006)
Supplement: Document S2. Article plus Supplemental Information [file mmc2.pdf]

Neuron

Report

Compulsivity Reveals a Novel Dissociation between Action and Confidence

Highlights

- Action can be functionally dissociated from confidence
- Patients with obsessive-compulsive disorder (OCD) show exaggerated action updating
- Confidence in OCD is intact and reflects information that is not used to control action
- Degree of action-confidence dissociation correlates with symptom severity in OCD

Authors

Matilde M. Vaghi, Fabrice Luyckx, Akeem Sule, Naomi A. Fineberg, Trevor W. Robbins, Benedetto De Martino

Correspondence

matilde.vaghi@gmail.com (M.M.V.), benedettodemartino@gmail.com (B.D.M.)

In Brief

Vaghi, Luyckx et al. provide evidences of a novel dissociation between confidence and action in OCD patients with confidence accessing information that is not used to guide action.

Vaghi et al., 2017, Neuron *96*, 348–354
October 11, 2017 © 2017 The Author(s). Published by Elsevier Inc.
http://dx.doi.org/10.1016/j.neuron.2017.09.006

Neuron
Report

Compulsivity Reveals a Novel Dissociation between Action and Confidence

Matilde M. Vaghi,[1,2,7,8,*] Fabrice Luyckx,[1,3,7] Akeem Sule,[4] Naomi A. Fineberg,[5] Trevor W. Robbins,[1,2] and Benedetto De Martino[6,*]
[1]Department of Psychology, University of Cambridge, Cambridge CB2 3EB, UK
[2]Behavioural and Clinical Neuroscience Institute (BCNI), University of Cambridge, Cambridge CB2 3EB, UK
[3]Department of Experimental Psychology, University of Oxford, Oxford OX2 6BW, UK
[4]Cumbria Partnership NHS Foundation Trust, NHS, Penrith, Cumbria CA11 7QQ, UK
[5]Hertfordshire Partnership University NHS Foundation Trust and University of Hertfordshire, Hertfordshire AL8 6HG, UK
[6]Institute of Cognitive Neuroscience, University College of London, Alexandra House, 17-19 Queen Square, London WC1N 3AR, UK
[7]These authors contributed equally
[8]Lead Contact
*Correspondence: matilde.vaghi@gmail.com (M.M.V.), benedettodemartino@gmail.com (B.D.M.)
http://dx.doi.org/10.1016/j.neuron.2017.09.006

SUMMARY

Confidence and actions are normally tightly interwoven—if I am sure that it is going to rain, I will take an umbrella—therefore, it is difficult to understand their interplay. Stimulated by the ego-dystonic nature of obsessive-compulsive disorder (OCD), where compulsive actions are recognized as disproportionate, we hypothesized that action and confidence might be independently updated during learning. Participants completed a predictive-inference task designed to identify how action and confidence evolve in response to surprising changes in the environment. While OCD patients (like controls) correctly updated their confidence according to changes in the environment, their actions (unlike those of controls) mostly disregarded this knowledge. Therefore, OCD patients develop an accurate, internal model of the environment but fail to use it to guide behavior. Results demonstrated a novel dissociation between confidence and action, suggesting a cognitive architecture whereby confidence estimates can accurately track the statistic of the environment independently from performance.

INTRODUCTION

Intelligent agents have to act on incomplete and fragmented information. Typically, incoming information is processed to reduce uncertainty so as to make more accurate inferences about the causal structure of the environment (Knill and Pouget, 2004; O'Reilly et al., 2012). Subjects are generally able to learn from experience, and often actions are dictated by subjects' beliefs acquired flexibly through this inference process. The strength of belief (or "confidence") is generally tightly coupled to behavior—I will study more for an exam if I am in doubt about my level of knowledge. Bayesian accounts of learning suggest that current levels of uncertainty in the estimate of the action's value influence behavior (Behrens et al., 2007; Nassar et al., 2010). In particular, the impact of information on behavior depends on the level of epistemic uncertainty (i.e., confidence) held by the agent. In other words, information is mostly influential when the agent is more uncertain about the environment. In contrast, when an agent has little uncertainty, it is less influenced by upcoming new evidence (Behrens et al., 2007; Nassar et al., 2010; Yu and Dayan, 2005). However, because of a tight link between behavior and confidence, it has been difficult to study how confidence and action evolve and interact during learning. In turn, obsessive-compulsive disorder (OCD) provides a paradigmatic example whereby the link between strength of beliefs and action can be disrupted. Compulsive rituals, such as hand washing, are called ego-dystonic, since patients recognize them as disproportionate and excessive but nevertheless cannot stop performing them (Kozak and Foa, 1994). Therefore, we capitalized on this distinctive feature of the disorder to test opposing views about how subjective beliefs are accessed for metacognitive reports of confidence and action control: specifically, are they accessed in parallel or is confidence necessarily informed by action monitoring?

At the same time, we sought to provide a novel computational insight in the etiology of this debilitating psychiatric condition.

RESULTS

Experimental Design

We studied 24 OCD patients and 25 matched controls (Table S1) on a modified predictive-inference task (McGuire et al., 2014; Nassar et al., 2010) (Figures 1A and 1B; STAR Methods). In each trial, participants were required to position a bucket on a circular ring to catch particles flying from the middle of the ring. After positioning the bucket (and before seeing where the particle would land), participants reported their degree of confidence in their prediction. During the task, subjects were required to gauge the value of a new piece of information

This is an open access article under the CC BY license (http://creativecommons.org/licenses/by/4.0/).

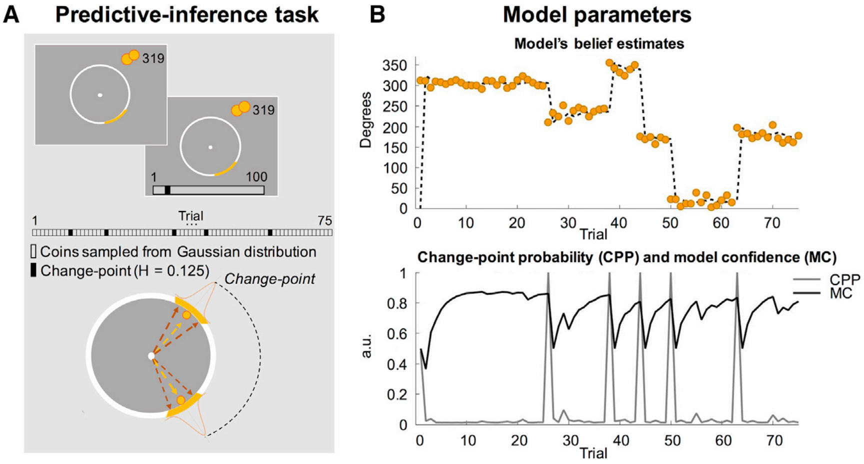

Figure 1. Diagram of the Predictive-Inference Task and Model Parameters

(A) On each trial, participants chose a position for the bucket (orange segment) and scored their confidence for their prediction on a bar appearing thereafter. One particle was then fired from the center of the big circle. Throughout each experimental block, particles were drawn from a Gaussian distribution. Mean of the distribution could change on any trial with a probability of 0.125 (H, hazard rate) determining a change in action-outcome contingencies in the environment (change-point).

(B) Top: example of a sequence of trials. Points mark the position at which particles landed on the big circle (0°–360°). The dotted line identifies the predictions of the quasi-optimal Bayesian model. Bottom: two theoretical factors, change-point probability (CPP) and model confidence (MC), jointly influence learning rate. When unexpected observations occur, CPP is high and MC attenuated.

and differentiate periods of time in which unexpected outcomes should be ignored as noise and those in which abrupt changes were likely to require updating of action and beliefs. In such unstable, dynamic environments, recent events are only informative in the presence of abrupt changes; meanwhile, during a stable period, most recent events are less informative, and decisions should be driven by averaging over the outcomes of many previous actions, i.e., historical information should be taken into account.

Behavioral Analyses

To investigate how new evidence collected in a noisy environment influences behavior (action) and confidence reports, we computed for each trial in each participant a spatial learning rate (LR, $\widehat{\alpha}$) expressed as the magnitude of change in the prediction (chosen bucket position from one trial to the next) as a fraction of the error made on the previous prediction (difference between chosen bucket position and position of new evidence). We then considered the same sequence of observations experienced by each participant and computed the behavior of a quasi-optimal Bayesian learning model (STAR Methods for the model specification; Nassar et al., 2010, 2012; McGuire et al., 2014). This model has no free parameters since it is not fitted to the participant behavior and provides a benchmark Bayesian model against which to compare participants' behavior. Following a procedure used previously (McGuire et al., 2014) and commonly used in model-based fMRI (O'Doherty et al., 2007), we then constructed regression models to test how participants' behavioral measures relate to different parameters of the benchmark Bayesian model with the aim to identify and quantify statistical differences between the two groups.

Learning Rate

In our task, participants' LRs ($\widehat{\alpha}$, Equation 1) were estimated on each trial by taking the ratio of bucket displacement and the spatial prediction error (McGuire et al., 2014; Nassar et al., 2010), measuring the extent to which each new outcome influenced subsequent prediction.

$$\widehat{\alpha}_t = \frac{\mathrm{b}_{t+1} - \mathrm{b}_t}{\widehat{\delta}_\mathrm{t}} \qquad \text{Equation 1}$$

$$\widehat{\delta}_\mathrm{t} = X_t - \mathrm{b}_t \qquad \text{Equation 2}$$

In Equations 1 and 2, $\mathrm{b_t}$ and $\mathrm{b_{t+1}}$ are the chosen bucket position (i.e., where participant positioned the bucket) for one trial and the next one. $\widehat{\delta}_\mathrm{t}$ is the spatial prediction error, which is the difference between the location of particle at trial t (X_t) and chosen bucket position at trial t ($\mathrm{b_t}$).

OCD patients were strongly influenced by recent outcomes as shown by their LR $(\widehat{\alpha})$ being significantly higher than the controls' (OCD, 0.52 ± 0.05; CTL, 0.31 ± 0.03; Welch two-tailed t test $t_{34} = -3.587$, $p = 0.001$) (Figure 2A). To investigate the effect of error magnitude (Figure 2B) on LR $(\widehat{\alpha})$, we divided the range of the spatial prediction error into quantiles and extracted data for controls and patients separately (Table S2; STAR Methods). Results suggested that LRs $(\widehat{\alpha})$ were highest after subjects made larger errors (effect of error magnitude, $F_{2,94} = 190.604$, $p < 0.001$), showing that both groups correctly monitored their actions, thus adapting their learning (Figure 2B). However, independently of error magnitude, LRs $(\widehat{\alpha})$ were systematically higher in patients than controls (effect of group, $F_{1,47} = 13.388$, $p < 0.001$) (Figure 2B), reflecting a more marked influence of a new outcome on subsequent prediction in the OCD group, regardless of the magnitude of error (groups pairwise comparisons low, $t_{26.627} = -3.267$, $p = 0.003$; medium, $t_{34.811} = -2.874$, $p = 0.007$; high, $t_{47} = -3.758$, $p = 0.0005$, Bonferroni corrected). Increased LRs $(\widehat{\alpha})$ in OCD patients did not correlate with medication dosage tested via Spearman correlation (OCD, $n = 24$, $\rho = -0.173$, $p = 0.418$). In addition, we tested the presence of an effect of medication by treating dosage as a categorical variable and testing the association with LRs $(\widehat{\alpha})$ divided into evenly spaced quantiles. Absence of an association was confirmed for multiple discretization of the LRs $(\widehat{\alpha})$ (all p values > 0.116). In the patient group, more marked updating of the bucket's position indexed by increased LRs $(\widehat{\alpha})$ was not associated with impulsivity as measured with the Barratt Impulsiveness Scale (Patton et al.,

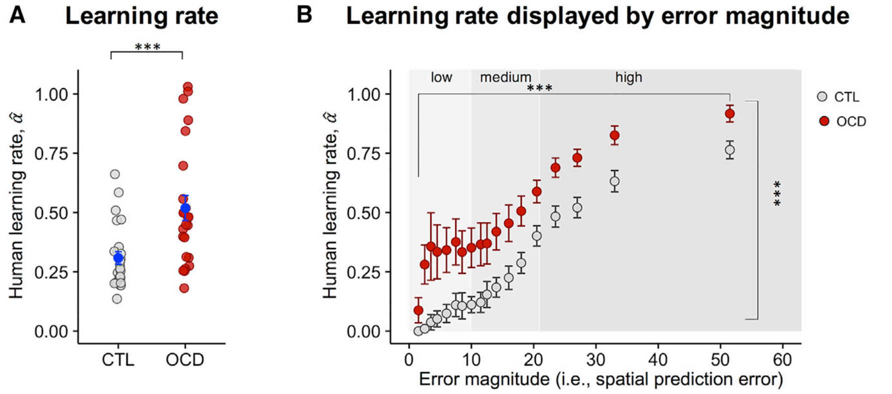

Figure 2. Learning Rates in OCD and Controls

(A) Learning rate (LR) for participants $(\hat{\alpha})$ (Equation 1). Patients showed significantly higher LRs $(\hat{\alpha})$ compared with controls. Dots represent individual subjects. Mean ± SEM are displayed in blue.

(B) LR for participants $(\hat{\alpha})$ (Equation 1) plotted as a function of the error magnitude (Equation 2). The distribution of the values of the spatial prediction error was divided in 20 quantiles (Table S2 for mean and SEM for each quantile). For visualization purposes, data from 18 quantiles are shown. Mean ± SEM are shown. Subjects tended to use variable LRs $(\hat{\alpha})$ spanning the entire allowed range, with higher LRs for higher spatial prediction error. However, LR $(\hat{\alpha})$ was higher in OCD patients, regardless of error magnitude. ***$p < 0.001$.

1995) (OCD, n = 24, Pearson's correlation, r = 0.162, p = 0.449). Therefore, increased LR $(\hat{\alpha})$ is likely driven by processes other than impulsiveness in OCD patients.

Dynamics of Learning and Confidence Update

To determine how participants' beliefs evolved over time, we compared the performance of our human participants to that of a quasi-optimal Bayesian learner carrying out the same task (Equation 3). The quasi-optimal Bayesian learner is a computationally parsimonious algorithmic implementation of an optimal Bayesian learner (Nassar et al., 2010 and McGuire et al., 2014 for a full comparison). In such simple tasks, it can achieve performance that is comparable to that of the optimal Bayesian learner at a fraction of the cost through a delta-rule type of learning in belief space.

$$B_{t+1} = B_t + \alpha_t \times \delta_t \qquad \text{Equation 3}$$

$$\alpha_t = \Omega_t + (1 - \Omega_t)(1 - \upsilon_t) \qquad \text{Equation 4}$$

$$\delta_t = X_t - B_t \qquad \text{Equation 5}$$

In Equation 3, B_t and B_{t+1} are the model's belief estimate about the mean of the distribution at trial t and t+1. The LR of the model (α) (Equation 4) is jointly influenced by the change-point probability (CPP, Ω) (Equation 6) and the model confidence (MC, υ) (Equation 8) (Figure 1B). δ (Equation 5) is the prediction error (PE), which is the discrepancy between the model belief estimate (B_t) and the location of the new sample (X_t). Note that unlike a full Bayesian model, in this Bayesian delta-rule model, beliefs are not represented by probability density but are point-like estimates that are iteratively updated like values in classic reinforcement learning models (McGuire et al., 2014; Nassar et al., 2010). Another feature of this algorithmic implementation is that there is a direct mapping between beliefs and action, which are not linked through a softmax function that converts values (or belief distributions) into action probabilities.

In response to change-points in the environment, when the information about the previous average position becomes irrelevant, the quasi-optimal Bayesian learner reacted by increasing its LR (α); subsequent to a change-point, as more evidence is accumulated from the same generative distribution, LR (α) steadily decreases, relying on integration of previous observations (Figure 3A). Our human participants followed a similar temporal dynamic in adjusting their LRs $(\hat{\alpha})$ (Figure 3B and Figure S1 for a close overlap at the trial-by-trial level between subject's bucket position computed in Equation 1 and model term B_t computed with Equation 3). However, in OCD patients, accumulation of more knowledge about the environment was unable to reduce LRs $(\hat{\alpha})$ sufficiently. In the extreme cases (patients with mean LR $(\hat{\alpha})$ = 1) behavior was exclusively driven by the last observed sample, ignoring all previously observed evidence. Therefore, LRs $(\hat{\alpha})$ were driven predominantly by the most recent outcomes disregarding previously experienced ones. Strikingly, and unlike action, the way in which confidence changed over time in OCD patients was indistinguishable from that of controls (Figure 3E) and closely resembled the quasi-optimal Bayesian learner's confidence (MC), which is bound to increase as more evidence is accumulated after a change-point (Figure 3D). This dissociation between action and confidence suggests that, in the OCD group, reports of confidence accurately reflected the increase in strength of beliefs following the accumulation of more evidence even when this evidence was underutilized for behavioral control.

To quantify and compare between-group differences in trial-wise adjustment in LRs $(\hat{\alpha})$, we performed linear regression analysis using the different parameters of the quasi-optimal Bayesian learner as predictors. Compared with controls, patients showed a stronger influence of PEs, indexing the tendency to update the bucket toward the most recent particle, in driving trial-wise adjustment in LRs $(\hat{\alpha})$ and reduced responses to abrupt changes in the environment indexed by CPP (PE: OCD, 0.57 ± 0.07; CTL, 0.26 ± 0.08; Wilcoxon rank-sum test, p = 0.018; CPP: OCD, 0.31 ± 0.07; CTL, 0.57 ± 0.06; Wilcoxon rank-sum test, p = 0.011) (Figure 3C; Table S3). There were no between-group differences for MC and for a categorical predictor indexing whether the previous trial resulted in a hit or miss (this was included in the regression since it has been proposed that OCD patients might exhibit differential sensitivity to reward and punishment; Endrass et al., 2011) (Table S3).

Using the same approach described above for action, we performed regression analysis to predict participants' confidence (Figure 3F). In contrast to the regression predicting participants'

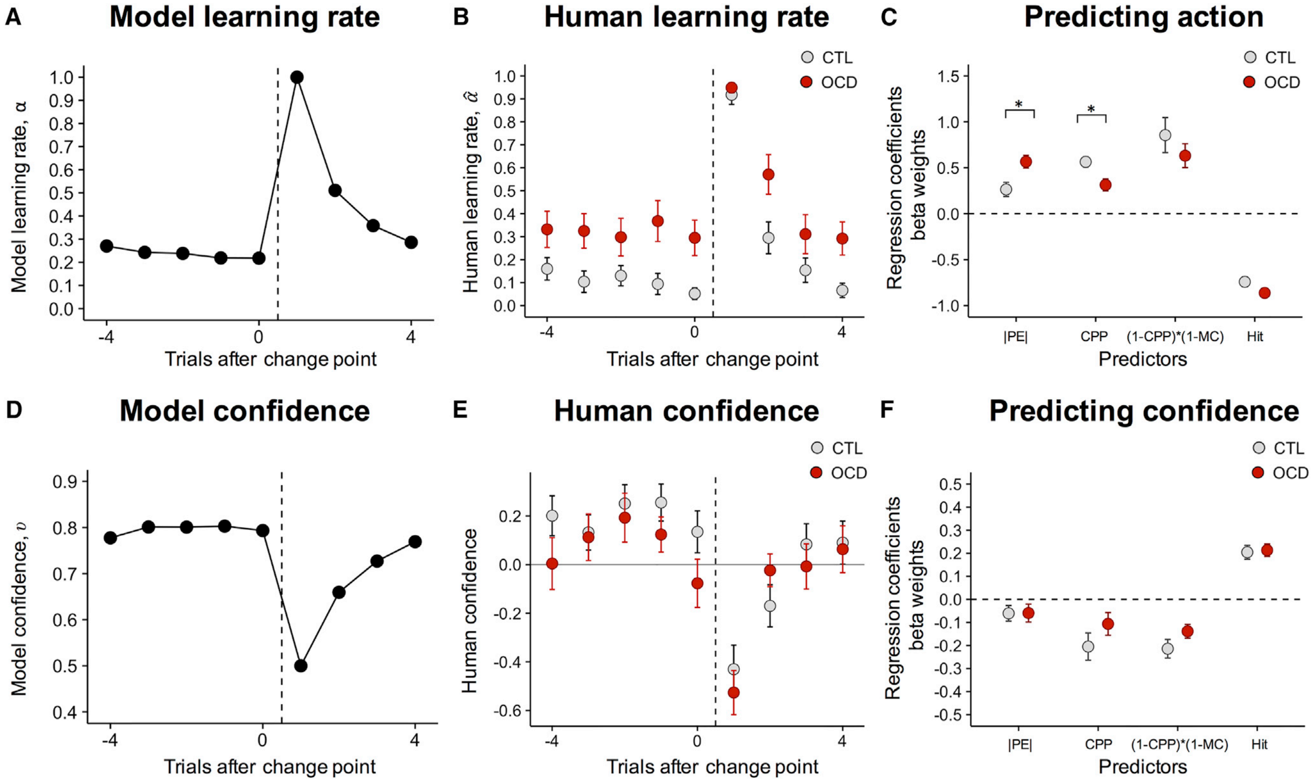

Figure 3. Regression-Based Analysis for Learning Rates and Confidence

(A and B) (A) Model learning rate (LR) (α) and (B) human LR ($\widehat{\alpha}$) aligned to change-points (vertical dashed line). LRs were highest after change-point trials and decayed thereafter. OCD patients showed increased LRs ($\widehat{\alpha}$) on trials before and after change-points.

(C) Regression analysis of behavioral data for human action, constructed similar to the update formula of the quasi-optimal Bayesian model, multiplying the LR ($\widehat{\alpha}$) by the absolute spatial prediction error ($|\widehat{\delta_t}|$).

(D and E) (D) Model confidence (υ) and (E) human confidence (*Z*-scored) aligned to change-points. The effects of confidence on LRs are greatest on the trials immediately after a change-point when confidence drops. Confidence recovers over several trials thereafter, with no between-group differences.

(F) Regression analysis of behavioral data for human confidence (*Z*-scored). Error bars represent SEM. Plotted predictors for action and confidence regressions correspond to absolute prediction error ($|\delta|$), change-point probability (Ω), model confidence (υ), and hit/miss categorical predictor. (A) (model learning rate) and (D) (model confidence) represent value of the first change-point. For (B) (human learning rate) and (E) (human confidence), all epochs were identified per subject where a change-point was preceded by five data points and followed by four. Group-level mean and SEM were calculated separately for controls and OCD patients. See also Table S3. $^*p < 0.05$.

action (Figure 3C), there were no between-group differences for confidence in any predictor (Figure 3F; Table S3). Therefore, OCD patients made full use of accumulated knowledge about the position of the particle to infer the underlying statistics of the environment and built accurate confidence estimates. This belief was nevertheless underutilized to control action, resulting in excessive reactivity to the most recent evidence and therefore elevated LRs ($\widehat{\alpha}$).

Such mismatching was formally tested by a new regression model in which action updating was predicted by confidence updating. In OCD patients, there was a weakened relationship between action control and metacognitive reports of confidence (OCD, 0.05 ± 0.01; CTL, 0.12 ± 0.02; Wilcoxon rank-sum test, $z = 2.690$, $p = 0.007$) (Figure 4A). Reduced coupling between action and belief was most prominent in more severely ill patients (OCD, $n = 24$, Pearson's correlation, $r = -0.426$, $p = 0.038$) (Figure 4B), thus relating inter-individual patient variability to symptom severity and suggesting that this computational deficit is a core feature of the multifaceted OCD psychiatric manifestation (Robbins et al., 2012; Stephan and Mathys, 2014).

DISCUSSION

In this study, we investigated how confidence interplays with action. We showed that both controls and OCD patients integrated historical information to update their confidence flexibly in keeping with the non-stationary features of the environment. More specifically, when the environment abruptly changed (i.e., change-point probability or CPP) confidence first decreased and then gradually increased again on subsequent trials when more evidence was accumulated as shown previously in healthy controls (Nassar et al., 2010, 2012). The dynamic by which confidence evolved over time closely matched that of a quasi-optimal Bayesian learner (McGuire et al., 2014; Nassar et al., 2010). Critically, we showed that while the estimation of confidence was intact in OCD patients, their actions largely

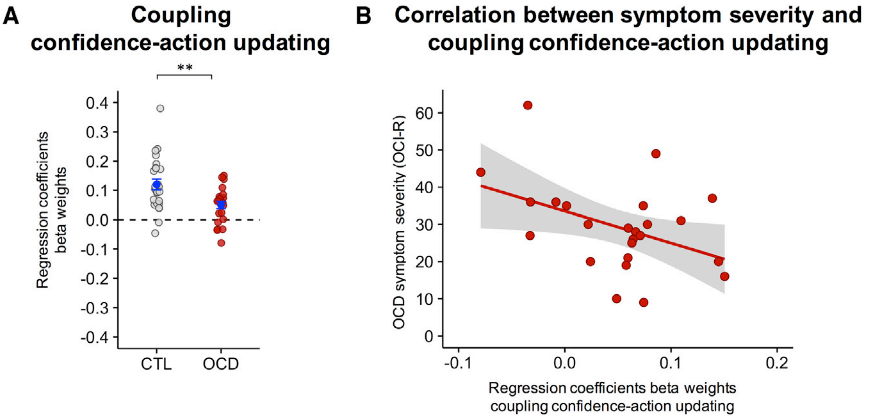

Figure 4. Uncoupling between Confidence and Action in OCD and Relationship with Symptom Severity

(A) Regression model whereby action updating (i.e., the absolute difference between where participants positioned bucket on trial t and t-1) is predicted by confidence updating (i.e., the absolute difference between *Z*-scored confidence reports on trial t and t-1). Dots represent individual subjects. Mean ± SEM are displayed in blue.

(B) Association between self-reported symptom severity and coupling confidence-action updating in patients. OCI-R, obsessive-compulsive inventory revised. Shaded gray area represents 95% confidence interval. $^{**}p < 0.01$.

disregarded this accumulated information. In other words, OCD patients constantly updated the position of the bucket in favor of the most recent outcomes rather than the average of the preceding trials. In extreme cases (participants with a LR $(\hat{\alpha})$ close to one), this behavior thus reflected abandonment of historical information acquired on previous trials, and as a consequence, the behavior was driven only by the last observation.

Hyperactive error signals have been linked to OCD and have been associated to the "not-just-right" experience, which forces these patients to monitor their behavior more carefully (Pitman, 1987). Here, however, higher LRs $(\hat{\alpha})$ in OCD were found even in the absence of an error and independently of its magnitude (Figure 2B), suggesting that elevated action update was not simply driven by response feedback. Instead, our computational analyses showed that, when updating their actions, OCD patients were more driven by prediction errors compared with controls.

More prominent action updating based on most recent outcomes in OCD is possibly due to an inability to take into account the broader context (i.e., history of outcomes during the experimental task) in order to build an internal map of the external world. These functions are generally attributed to model-based strategies exploited to generate goal-directed choices and held to produce cognitive predictions of future values based on representations of the environment, expectations, and prospective calculations (Daw et al., 2005). A weakness of this system in OCD might leave room for producing automatic stimulus-response habits. Development of action-outcome predictions and PE is heavily dependent on dopamine in the ventral striatum (VS) (O'Doherty et al., 2004). Blunted VS reactivity in response to reward has been shown several times in OCD (Figee et al., 2013; Marsh et al., 2015).

In addition, the anterior cingulate cortex (ACC) might underpin the behavioral adjustments required to perform our task. ACC has been shown to be critically involved in updating learning rates when circumstance in the environment are changing (Behrens et al., 2007; Kennerley et al., 2006; McGuire et al., 2014). Therefore, an intriguing possibility is that the exaggerated behavioral updating in OCD patients shown here might implicate a dysfunction in a frontostriatal loop involving the VS and the ACC (Haber and Behrens, 2014) and might possibly explain how deep brain stimulation successfully targets this frontostriatal circuit (Denys et al., 2010; Figee et al., 2013) in the treatment of OCD.

Furthermore, our work shows that the degree of uncoupling between confidence and action is correlated with the severity of the OCD symptomatology, showing that this uncoupling (that we report here, we believe, for the first time) might be at the core of the computational deficit that characterizes OCD. Previous work has proposed that OCD patients might suffer from a metacognitive deficit (Hauser et al., 2017; Hermans et al., 2008). Our results are consistent with a metacognitive deficit in OCD (i.e., patients' confidence reports were not tracking their actions) but suggest that OCD metacognitive deficits might not be triggered by an abnormal confidence response but by the uncoupling between action and subjective confidence ratings that we have isolated.

These findings might also help to distinguish between competing accounts of human cognition by clarifying the link between confidence and behavioral control. A prevailing view is that confidence is built from internal cues related to object-level processes and through monitoring one's own actions (Metcalfe and Shimamura, 1994) to support error detection (Yeung and Summerfield, 2012). In the context of signal-detection theory, it has been proposed that confidence is built from a subjective internal state (decision variable) that is influenced by sensory evidence. Confidence is then construed as the absolute distance between a decision variable and a criterion (Kepecs et al., 2008; Vickers, 1979). A similar logic underpins sequential sampling models with optional stopping (e.g., race models, drift diffusion models) in which confidence is computed from the state of the decision variable when the choice is emitted (De Martino et al., 2013; Fetsch et al., 2014; Kiani and Shadlen, 2009; Vickers, 1979). An exciting recent development of this approach suggests that confidence is also informed by parameters relating to the subject's own actions such as response latency (Fetsch et al., 2014; Kiani et al., 2014). Our findings nuance this view by showing that OCD patients can build accurate confidence estimates based on information that correctly reflects the statistics of their environment, even when their actions are not guided by this information. This finding has broader implications that go beyond OCD. Notably, the simple fact that this population is able to build accurate confidence estimates (integrating information over time) even if actions are not driven by this information (but only by the most recent samples) speaks against a general view in which actions *must* be monitored in order to build

accurate confidence reports. Based on these results, we therefore suggest a parallel coding scheme in which "beliefs" can *independently* control actions and give rise to a metacognitive sense of confidence.

The notion that confidence and performance can be dissociated is not entirely new. Recent studies have proposed that confidence, in order to become available for self-report, needs to be "read out" by an anatomically distinct network in the prefrontal cortex; during this process, information can be corrupted by further noise that does not affect action (De Martino et al., 2013; Fleming et al., 2012; Insabato et al., 2010; Maniscalco and Lau, 2012). Moreover, confidence estimates might be modified by further information processed in the time elapsing between when an action is performed and when a confidence rating is reported (Fleming, 2016; Moran et al., 2015; Navajas et al., 2016; Resulaj et al., 2009) (for a comprehensive review, see Fleming and Daw, 2017). However, all these accounts suggest that such a dissociation is driven by the fact that actions correctly reflect the information available when the choice is elicited, possibly incorporating extra information that is inaccessible to metacognitive reports of confidence (Meyniel et al., 2015). In this contribution, inspired by the distinctive ego-dystonic nature of OCD, we were able to demonstrate a novel dissociation in which actions can underutilize (or fail to access) information about the environment that is fully available to the decision maker and accessible to reports of confidence. We further suggest that a correct coupling between actions and confidence might be critical for fully functional behavior. In conclusion, we hope that this novel dissociation might help to constrain models of how beliefs are used to control behavior in the healthy brain while at the same time informing our understanding of the mechanisms underpinning OCD.

STAR★METHODS

Detailed methods are provided in the online version of this paper and include the following:

- KEY RESOURCES TABLE
- CONTACT FOR REAGENT AND RESOURCE SHARING
- EXPERIMENTAL MODEL AND SUBJECT DETAILS
  - Human Subjects
- METHOD DETAILS
  - Behavioral Task
  - Procedure
  - Computational Model
- QUANTIFICATION AND STATISTICAL ANALYSIS
  - Learning Rate Computation: Humans and Model
  - Behavioral Analysis
  - Regression Model
- DATA SOFTWARE AVAILABILITY
  - Data and Code Availability

SUPPLEMENTAL INFORMATION

Supplemental Information includes four figures and four tables and can be found with this article online at http://dx.doi.org/10.1016/j.neuron.2017.09.006.

AUTHOR CONTRIBUTIONS

Conceptualization, M.M.V., F.L., T.W.R., and B.D.M.; Methodology, M.M.V., F.L., and B.D.M.; Model implementation, F.L.; Data acquisition, M.M.V.; Data analysis, M.M.V. and F.L.; Resources, N.A.F. and A.S.; Writing – Review and Editing, M.M.V., F.L., N.A.F., A.S., T.W.R., and B.D.M.; Supervision, B.D.M.; Funding acquisition, T.W.R.

ACKNOWLEDGMENTS

B.D.M. was supported by Wellcome Trust and Royal Society (Sir Henry Dale Fellowship 102612/A/13/Z). T.W.R. was supported by Wellcome Trust (Senior Investigator Award 104631/Z/14/Z). M.M.V. is supported by a Pinsent Darwin Scholarship in Mental Pathology and an Angharad Dodds John Bursary in Mental Health and Neuropsychiatry. We also thank Steve Fleming for his comments on a previous draft of this article.

Received: April 20, 2017
Revised: August 1, 2017
Accepted: September 7, 2017
Published: September 28, 2017

REFERENCES

Behrens, T.E.J., Woolrich, M.W., Walton, M.E., and Rushworth, M.F.S. (2007). Learning the value of information in an uncertain world. Nat. Neurosci. *10*, 1214–1221.

Daw, N.D., Niv, Y., and Dayan, P. (2005). Uncertainty-based competition between prefrontal and dorsolateral striatal systems for behavioral control. Nat. Neurosci. *8*, 1704–1711.

De Martino, B., Fleming, S.M., Garrett, N., and Dolan, R.J. (2013). Confidence in value-based choice. Nat. Neurosci. *16*, 105–110.

Denys, D., Mantione, M., Figee, M., van den Munckhof, P., Koerselman, F., Westenberg, H., Bosch, A., and Schuurman, R. (2010). Deep brain stimulation of the nucleus accumbens for treatment-refractory obsessive-compulsive disorder. Arch. Gen. Psychiatry *67*, 1061–1068.

Endrass, T., Kloft, L., Kaufmann, C., and Kathmann, N. (2011). Approach and avoidance learning in obsessive-compulsive disorder. Depress. Anxiety *28*, 166–172.

Fetsch, C.R., Kiani, R., and Shadlen, M.N. (2014). Predicting the accuracy of a decision: a neural mechanism of confidence. Cold Spring Harb. Symp. Quant. Biol. *79*, 185–197.

Figee, M., Luigjes, J., Smolders, R., Valencia-Alfonso, C.-E., van Wingen, G., de Kwaasteniet, B., Mantione, M., Ooms, P., de Koning, P., Vulink, N., et al. (2013). Deep brain stimulation restores frontostriatal network activity in obsessive-compulsive disorder. Nat. Neurosci. *16*, 386–387.

Fleming, S.M. (2016). Changing our minds about changes of mind. eLife *5*, e14790.

Fleming, S.M., and Daw, N.D. (2017). Self-evaluation of decision-making: a general Bayesian framework for metacognitive computation. Psychol. Rev. *124*, 91–114.

Fleming, S.M., Huijgen, J., and Dolan, R.J. (2012). Prefrontal contributions to metacognition in perceptual decision making. J. Neurosci. *32*, 6117–6125.

Foa, E.B., Huppert, J.D., Leiberg, S., Langner, R., Kichic, R., Hajcak, G., and Salkovskis, P.M. (2002). The Obsessive-Compulsive Inventory: development and validation of a short version. Psychol. Assess. *14*, 485–496.

Goodman, W.K., Price, L.H., Rasmussen, S.A., Mazure, C., Fleischmann, R.L., Hill, C.L., Heninger, G.R., and Charney, D.S. (1989). The Yale-Brown Obsessive Compulsive Scale. I. Development, use, and reliability. Arch. Gen. Psychiatry *46*, 1006–1011.

Haber, S.N., and Behrens, T.E.J. (2014). The neural network underlying incentive-based learning: implications for interpreting circuit disruptions in psychiatric disorders. Neuron *83*, 1019–1039.

Hauser, T.U., Allen, M., Rees, G., and Dolan, R.J.; NSPN Consortium (2017). Metacognitive impairments extend perceptual decision making weaknesses in compulsivity. Sci. Rep. *7*, 6614.

Hermans, D., Engelen, U., Grouwels, L., Joos, E., Lemmens, J., and Pieters, G. (2008). Cognitive confidence in obsessive-compulsive disorder: distrusting perception, attention and memory. Behav. Res. Ther. *46*, 98–113.

Insabato, A., Pannunzi, M., Rolls, E.T., and Deco, G. (2010). Confidence-related decision making. J. Neurophysiol. *104*, 539–547.

Kennerley, S.W., Walton, M.E., Behrens, T.E.J., Buckley, M.J., and Rushworth, M.F.S. (2006). Optimal decision making and the anterior cingulate cortex. Nat. Neurosci. *9*, 940–947.

Kepecs, A., Uchida, N., Zariwala, H.A., and Mainen, Z.F. (2008). Neural correlates, computation and behavioural impact of decision confidence. Nature *455*, 227–231.

Kiani, R., and Shadlen, M.N. (2009). Representation of confidence associated with a decision by neurons in the parietal cortex. Science *324*, 759–764.

Kiani, R., Corthell, L., and Shadlen, M.N. (2014). Choice certainty is informed by both evidence and decision time. Neuron *84*, 1329–1342.

Knill, D.C., and Pouget, A. (2004). The Bayesian brain: the role of uncertainty in neural coding and computation. Trends Neurosci. *27*, 712–719.

Kozak, M.J., and Foa, E.B. (1994). Obsessions, overvalued ideas, and delusions in obsessive-compulsive disorder. Behav. Res. Ther. *32*, 343–353.

Maniscalco, B., and Lau, H. (2012). A signal detection theoretic approach for estimating metacognitive sensitivity from confidence ratings. Conscious. Cogn. *21*, 422–430.

Marsh, R., Tau, G.Z., Wang, Z., Huo, Y., Liu, G., Hao, X., Packard, M.G., Peterson, B.S., and Simpson, H.B. (2015). Reward-based spatial learning in unmedicated adults with obsessive-compulsive disorder. Am. J. Psychiatry *172*, 383–392.

McGuire, J.T., Nassar, M.R., Gold, J.I., and Kable, J.W. (2014). Functionally dissociable influences on learning rate in a dynamic environment. Neuron *84*, 870–881.

Metcalfe, J., and Shimamura, A.P. (1994). Knowing about Knowing (The MIT Press).

Meyniel, F., Sigman, M., and Mainen, Z.F. (2015). Confidence as bayesian probability: from neural origins to behavior. Neuron *88*, 78–92.

Montgomery, S.A., and Asberg, M. (1979). A new depression scale designed to be sensitive to change. Br. J. Psychiatry *134*, 382–389.

Moran, R., Teodorescu, A.R., and Usher, M. (2015). Post choice information integration as a causal determinant of confidence: novel data and a computational account. Cognit. Psychol. *78*, 99–147.

Nassar, M.R., Wilson, R.C., Heasly, B., and Gold, J.I. (2010). An approximately Bayesian delta-rule model explains the dynamics of belief updating in a changing environment. J. Neurosci. *30*, 12366–12378.

Nassar, M.R., Rumsey, K.M., Wilson, R.C., Parikh, K., Heasly, B., and Gold, J.I. (2012). Rational regulation of learning dynamics by pupil-linked arousal systems. Nat. Neurosci. *15*, 1040–1046.

Nassar, M.R., Bruckner, R., Gold, J.I., Li, S.-C., Heekeren, H.R., and Eppinger, B. (2016). Age differences in learning emerge from an insufficient representation of uncertainty in older adults. Nat. Commun. *7*, 11609.

Navajas, J., Bahrami, B., and Latham, P.E. (2016). Post-decisional accounts of biases in confidence. Curr. Opin. Behav. Sci. *11*, 55–60.

O'Doherty, J., Dayan, P., Schultz, J., Deichmann, R., Friston, K., and Dolan, R.J. (2004). Dissociable roles of ventral and dorsal striatum in instrumental conditioning. Science *304*, 452–454.

O'Doherty, J.P., Hampton, A., and Kim, H. (2007). Model-based fMRI and its application to reward learning and decision making. Ann. N Y Acad. Sci. *1104*, 35–53.

O'Reilly, J.X., Jbabdi, S., and Behrens, T.E.J. (2012). How can a Bayesian approach inform neuroscience? Eur. J. Neurosci. *35*, 1169–1179.

Patton, J.H., Stanford, M.S., and Barratt, E.S. (1995). Factor structure of the barratt impulsiveness scale. J. Clin. Psychol. *51*, 768–774.

Pitman, R.K. (1987). A cybernetic model of obsessive-compulsive psychopathology. Compr. Psychiatry *28*, 334–343.

Resulaj, A., Kiani, R., Wolpert, D.M., and Shadlen, M.N. (2009). Changes of mind in decision-making. Nature *461*, 263–266.

Robbins, T.W., Gillan, C.M., Smith, D.G., de Wit, S., and Ersche, K.D. (2012). Neurocognitive endophenotypes of impulsivity and compulsivity: towards dimensional psychiatry. Trends Cogn. Sci. *16*, 81–91.

Sheehan, D.V., Lecrubier, Y., Sheehan, K.H., Amorim, P., Janavs, J., Weiller, E., Hergueta, T., Baker, R., and Dunbar, G.C. (1998). The Mini-International Neuropsychiatric Interview (M.I.N.I.): the development and validation of a structured diagnostic psychiatric interview for DSM-IV and ICD-10. J. Clin. Psychiatry *59* (*Suppl 20*), 22–33, quiz 34–57.

Spielberger, C.D. (1983). Manual for the State-Trait Anxiety Inventory (STAI) (Consulting Psychologists Press).

Stephan, K.E., and Mathys, C. (2014). Computational approaches to psychiatry. Curr. Opin. Neurobiol. *25*, 85–92.

Stephan, K.E., Penny, W.D., Daunizeau, J., Moran, R.J., and Friston, K.J. (2009). Bayesian model selection for group studies. Neuroimage *46*, 1004–1017.

Vickers, D. (1979). Decision Processes in Visual Perception (Academic Press).

Yeung, N., and Summerfield, C. (2012). Metacognition in human decision-making: confidence and error monitoring. Philos. Trans. R. Soc. Lond. B Biol. Sci. *367*, 1310–1321.

Yu, A.J., and Dayan, P. (2005). Uncertainty, neuromodulation, and attention. Neuron *46*, 681–692.

STAR★METHODS

KEY RESOURCES TABLE

REAGENT or RESOURCE	SOURCE	IDENTIFIER
Deposited Data		
Behavioral data	This paper	https://github.com/BDMLab/Vaghi_Luyckx_et_al_2017 and https://doi.org/10.17863/CAM.13236 for raw, unprocessed behavioral data
Software and Algorithms		
MATLAB	MathWorks	Matlab_R2015b
R	R Development Core Team (2008)	http://www.R-project.org.
Custom code (experiment, model, analyses)	This paper	https://github.com/BDMLab/Vaghi_Luyckx_et_al_2017

CONTACT FOR REAGENT AND RESOURCE SHARING

All resources, including data and codes used for the analyses on this paper, are publicly available (see Data Software Availability and Key Resource Table). Further information and requests for resource sharing should be directed to and will be fulfilled by the Lead Contact, Matilde M. Vaghi (matilde.vaghi@gmail.com).

EXPERIMENTAL MODEL AND SUBJECT DETAILS

Human Subjects

The study included 49 participants, consisting of 24 patients with Obsessive-Compulsive Disorder (OCD) and 25 healthy volunteers matched for gender, age, and estimated verbal IQ using the National Adult Reading Test (data from one participant were not included being Spanish mother tongue) (Table S1). Control subjects were recruited from the community; none of them were on psychiatric medication and they never suffered from a psychiatric disorder. Patients were recruited through clinical referral from local psychiatric and psychological services or local advertisement. We ensured that patients met criteria for OCD diagnosis and did not suffer from any current comorbidity. When recruitment was conducted through advertisement, a consultant psychiatrist (N.A.F. or A.S.) made DSM-5 diagnoses using an extended clinical interview, supplemented by the Mini International Neuropsychiatric Interview (Sheehan et al., 1998). Exclusion criteria for all participants were current substance dependence, head injury and current depression, indexed by Montgomery-Åsberg Depression Rating Scale (MADRS) (Montgomery and Asberg, 1979) exceeding 16 during screening. OCD patients were not enrolled in the study if they scored less than 12 on the Yale-Brown Obsessive-Compulsive Scale (Y-BOCS) (Goodman et al., 1989) and if they reported hoarding symptomatology. Self-reported measures of anxiety were collected using the State-Trait Anxiety Inventory (STAI) (Spielberger, 1983); and, in addition to Y-BOCS scores, self-reported measures of OCD symptomatology were collected using the Obsessive-Compulsive Inventory-Revised (OCI-R) (Foa et al., 2002). OCD patients reported higher levels of depressive symptoms and anxiety, though well below clinical threshold (Table S1). Sixteen of the 24 patients were taking a stable dose of serotonin reuptake inhibitors (SSRIs) medication for a minimum of 8 weeks prior to taking part in the study. Eight unmedicated patients were included in the study, being either drug-naive or off medication for at least 8 weeks prior taking part of the study. Due to insufficient power, we were limited in the possibility of making direct comparison between medicated and unmedicated patients for the relevant behavioral measures. However, to test for a potential role of medication on behavioral measures of interest, and to overcome this limitation, we divided patients into 4 categories according to medication dosages considered to represent clinical equivalents. The study was approved by the NHS East of England, Cambridge Central Research Ethics Committee. Participants were reimbursed for their time and informed consent was obtained prior to participation. Participants completed two other behavioral tasks, unrelated to the present study. No statistical methods were used to pre-determine sample size but our sample sizes are similar to those generally employed in the field.

METHOD DETAILS

Behavioral Task

The task consisted of a particle released from the center of a large circle, which participants were asked to catch with a bucket (orange segment in Figure 1A) placed at the edge of the circle. After they positioned the bucket, participants gave a score between 1 and 100 on how confident they were the particle would land in their bucket. The particle's location was determined on each trial by

sampling a Gaussian distribution; thus particles usually landed in the same location with small variations only determined by noise. This procedure introduced uncertainty into the estimation. The mean of this distribution usually remained stable over a block of trials but changed at random intervals (*change-points*) when it was resampled from a uniform distribution, thus requiring the participant to form a new belief about the mean of the new generative Gaussian distribution (Figures 1A and 1B).

A trial started with participants choosing a location for the bucket. The bucket could be moved around with the rotary controller. When a location was chosen, they confirmed by pressing the spacebar. After 150 ms a confidence bar would appear below the big circle, where participants could indicate how confident they were the particle would land in the bucket. The confidence pointer would always start on a random score between 25 and 75, so participants were forced to move the pointer even when their bucket position had not changed. The confidence pointer could be moved around with the rotary controller and a decision was confirmed by pressing the spacebar. A particle was released 150 ms after a reporting of confidence. If the particle landed within the boundaries of the bucket, the bucket would turn white for 1 frame, creating a short flash. Subsequently the center dot would turn green for 800 ms and a consonant tone was played simultaneously for 400 ms. Alternatively, when the particle missed the bucket, the center dot turned red and a dissonant tone was played for 400 ms. Catching the particle resulted in a gain of 10 points, while missing a particle resulted in a loss of 10 points, thus payment was fixed throughout the task and we did not incentivize confidence. Each block started with 0 points and their total score was the sum of points gathered by the end of each block. Payment was performance contingent: the more points participants gathered, the more money they earned at the end up, to a maximum of £ 5.

Before the start of the experiment, participants were shown the layout of the experiment while being instructed on the purpose of the experiment. Participants then completed 20 practice trials that were excluded from any analysis and did not count for their final score. The actual experiment lasted for 4 blocks of 75 particles, thus each block consisted of 75 trials (Figures 1A and 1B). There was no time limit during a trial, but participants were instructed to act as quickly and accurately as possible. In total, the full experiment lasted around 18 min.

Procedure

The experiment was programmed in MATLAB (MathWorks) using Psychtoolbox 3. Input was given through the Griffin PowerMate USB rotary controller - to comply with our circular design - and a spacebar was pressed for confirmation responses.

For each participant, particles were drawn from a normal Gaussian distribution with a low fixed standard deviation of 12. The mean of the distribution could change on any trial with a probability of 0.125 (hazard rate, change-point, Figure 1A), drawn from a uniform distribution *U(1,360)*.

A white circle (ø = 500 px) with a dot (ø = 16 px) in the center was displayed permanently in the middle of the screen on a gray background. The so-called 'bucket' was designed as a portion of the circle displaced outwardly, moving along the outside border of the big circle. The bucket spanned 3 times the SD of the generative distribution, covering an area of 36 possible locations. Particles were represented by a yellow dot with a diameter of 16 pixels that flew from the center of the big circle to the edge at a speed of 30 frames per second (approx. 500 ms). A white confidence bar (w = 500px, h = 20px) appeared below the big circle, with a black pointer (w = 5 px) starting at a random location between a score of 25 and 75. The numbers 1 and 100 below the left and right corner respectively of the confidence bar indicated the range of possible scores. The points accumulated during the running block was presented in the top right corner, so participants could keep track of their performance. After each block, the total score was also displayed. Data collection and analysis were not performed blind to the conditions of the experiment. See quantification and statistical analysis for inclusion and exclusion criteria of any data.

Computational Model

Our model was an implementation of the reduced quasi-optimal Bayesian observer from McGuire et al. (2014), with slight adjustments to work with our circular data. The model, originally proposed by Nassar et al. (2010), attempts to approximate the behavior of a full Bayesian model, without the computational complexity of having to search through the full state space. It employs a simple delta rule to estimate a new belief estimate about the environment on every trial.

In our case, the model's belief estimate B_t about the environment is equal to a point estimation of the mean of the current normal Gaussian distribution from which samples are drawn (Equation 3). The learning rate (α) (Equation 4) determines how much the new sample will influence the model's belief estimate. If $\alpha_t = 0$, the model will not alter its current belief estimate at all, but when $\alpha_t = 1$, the most recent outcome will determine the updated belief estimate entirely.

In contrast to common reinforcement learning models where learning rate is fixed, the model employs a dynamic learning rate that can change on any trial (Equation 4). This allows the model to take into account large changes in the environment - indicating a change of location - and disregard outliers when beliefs estimates about the mean are well established. Learning rate (α) thus consists of two components that are also updated on each trial. The first component is the change-point probability (CPP) Ω_t (Equation 6), which indicates the model's suspicion that a change in location has occurred, and the second is the model confidence (MC) υ_t (Equation 8), which takes into account the uncertainty arising from the imprecise estimation of the mean. In other words, learning rate (α) will be high when the model assumes a change in location has occurred or when the model is uncertain about the mean.

CPP in Equation 6 is constructed as the relative likelihood that a new sample is drawn from the same Gaussian distribution (N), centered around the current belief estimate B_t of the model, or alternatively from a uniform distribution (U) over all 360 possible locations.

$$\Omega_t = \frac{U(X_t\,|\,1,360)H}{U(X_t\,|\,1,360)H + N(X_t\,|\,B_t,\sigma_t^2)(1-H)} \quad \text{Equation 6}$$

H is the hazard rate, the probability that the mean of the distribution has changed. When comparing behavior to the quasi-optimal model, we set H equal to the hazard rate of the experiment (H = 0.125, but see Figures S3 and S4). CPP will be close to 1 when the probability of the sample coming from the uniform distribution is greater than the probability of it coming from the normal distribution (i.e., a surprising outcome). σ^2_t is the estimated variance of the predictive distribution (not to be confused with σ^2_N, the variance of the generative Gaussian distribution).

$$\sigma_t^2 = \sigma_N^2 + \frac{(1-\upsilon_t)\sigma_N^2}{\upsilon_t} \quad \text{Equation 7}$$

This formula (Equation 7) has been validated in a previous paper (Nassar et al., 2012). It consists of two terms: the variance of the generative Gaussian distribution σ^2_N and the same variance but modulated by model confidence υ_t. Because σ^2_t is modulated by υ_t, the variance on trials immediately after a change-point will be larger than the generative variance, but will slowly decay toward the generative variance again. As a result, new samples drawn shortly after a change-point will be interpreted more conservatively, i.e., the model will be less inclined to assume a new change-point under the conditional distribution.

In contrast to the other model variables, MC in Equation 8 is computed for the *subsequent* trial. It takes into account the uncertainty arising from the imprecise estimation of the mean, opposed to uncertainty arising from noise (i.e., σ^2_N).

$$\upsilon_{t+1} = \frac{\Omega_t\sigma_N^2 + (1-\Omega_t)(1-\upsilon_t)\sigma_N^2 + \Omega_t(1-\Omega_t)(\delta_t\upsilon_t)^2}{\Omega_t\sigma_N^2 + (1-\Omega_t)(1-\upsilon_t)\sigma_N^2 + \Omega_t(1-\Omega_t)(\delta_t\upsilon_t)^2 + \sigma_N^2} \quad \text{Equation 8}$$

Analytically model confidence is (1-RU), the additive inverse of relative uncertainty (RU), from McGuire et al. (2014). For comparison purposes with humans' reported confidence we chose to represent this parameter as a confidence measure. The two models are mathematically identical. The first term of the nominator computes the variance when a change-point is assumed to have occurred ($\upsilon_t = 0.5$), while the second term is conditional on no change-point (slowly decaying uncertainty). The third term of the nominator reflects a rise in uncertainty when the model is not sure whether a change-point has in fact occurred. The same three terms are repeated in the denominator, with an added variance term reflecting the uncertainty arising from noise.

QUANTIFICATION AND STATISTICAL ANALYSIS

Learning Rate Computation: Humans and Model

For each participant, on each trial, LR ($\widehat{\alpha}_t$) was computed according to the formula in Equation 1. Namely, LR ($\widehat{\alpha}_t$) corresponded to the ratio of difference between chosen bucket position (i.e., where participant positioned the bucket) from one trial to the next and the spatial prediction error (i.e., the difference between the location of particle at trial t and chosen bucket position at trial t). Therefore, LR ($\widehat{\alpha}$) was empirically derived for each individual subject on each trial based on the experimental data. For the quasi-optimal Bayesian model, learning rate on each trial (α_t) was computed according to the formula in Equation 4. Computation of CPP was construed as the relative likelihood that a new sample is drawn from the same Gaussian distribution as shown in Equations 6 and 7. Therefore, the position of the new sample at a given trial X_t corresponded to the empirical data (i.e., where the particle landed). The belief estimate of the model for the first trial (B_t, t = 1) was initialized as 0. MC was implemented according to Equation 8 and initialized for the first trial (υ_t, t = 1) as 0.5 (i.e., the same value assigned for when a change-point was assumed to have occurred). Values of the hazard rate (H = 0.125) and variance of the generative Gaussian distribution ($\sigma^2_N = 12$) were fixed as described in previous section.

Behavioral Analysis

All analyses were conducted in MATLAB (MathWorks) with used in-house scripts and functions and R version 3.3.1 via RStudio version 0.99.878 (https://www.r-project.org/). All statistical tests were two-sided, and parametric or nonparametric tests applied as needed according to assumptions of the specific statistical test chosen.

We excluded trials from the analysis where the estimated LR ($\widehat{\alpha}$) exceeded the 99th percentile (calculated separately for control and patient group) or where the spatial prediction error (i.e., $\widehat{\delta}_t = X_t - b_t$, Equation 2) was equal to 0. Those were respectively thought to be due to processes other than error-driven learning or provided no information about error-driven learning (Nassar et al., 2016). Consequently, we omitted 3.1% of all trials. All participants (OCD, n = 24; CTL, n = 25) were included for the statistical analyses if not reported otherwise.

All confidence measures were z-scored within subjects to make comparison between groups possible. Additional analyses were performed in order to ensure that there were no differences between the groups that may have arisen by chance. First, we computed the number of change-points occurring for each subject and compared between groups. There were no differences in the total number of change-points occurring (OCD, 42.70 ± 1.16; Controls, 41.56 ± 1.98; $t_{47} = 0.760$, $p = 0.451$). Second, because

change-points might have occurred at any point, we identified periods of stability (periods in between change-points). There were no groups differences in number of trials occurring between consecutive change-points (OCD, 6.95 ± 0.20; Controls, 7.07 ± 0.16; t_{47} = 0.491, p = 0.625). Third, because position of change-points was re-drawn from a Gaussian distribution, particles at change-points might have landed on any location of the circumference. The distance between the position of the particle preceding a change-point and the position of the particle at a change-point was not different between groups (OCD, 83.39 ± 1.20; Controls, 80.41 ± 2.08; t_{47} = 1.228, p = 0.226). Fourth, because of the jittering during periods of stability (periods in between change-points) particles might have landed in the proximities of the mean. As expected, given the fixed standard deviation of the Gaussian distribution, there were no significant differences in the position of the particles during periods of stability (OCD, 13.64 ± 0.13; Controls, 13.74 ± 0.18; $t_4$7 = 0.406, p = 0.686).

LR $(\widehat{\alpha})$ was compared across groups (Figure 2A) using Welch's two-tailed t test adjusting degrees of freedom to account for unequal variances.

For data in Figure 2B we computed, for each participant, for each trial, the error magnitude (i.e., $\widehat{\delta}_t = X_t\text{-}b_t$, Equation 2 corresponding to the difference between the location of particle at trial t and chosen bucket position at trial t). The distribution of the values of the spatial prediction error was divided into 20 quantiles. The MATLAB function quantile was used dividing the frequency distribution of the spatial prediction error into 20 groups (see Table S2 for mean and SEM for each quantile). As a result, the quantiles were not equally spaced but contained the same fraction of the total data distribution. For each quantile, we computed the mean LR $(\widehat{\alpha})$ separately in controls and patients. Only for visualization purposes, data from the first 18 quantiles are shown in Figure 2B (but see Table S2 for mean and SEM of each quantile). For statistical analysis, the distribution of the values of the spatial prediction error was divided in 3 quantiles (i.e., low, medium, and high error magnitude), data extracted for controls and OCD for each of the three quantiles, and statistical analysis performed. A mixed two factor within subject design was used to analyze the data having group as the between subject factor and magnitude of error as the within subject. "Ez" R package yielding ANOVA results and assumptions check (Mauchly's test for sphericity, sphericity corrections and Levene's test for homogeneity of variance) was employed. Sphericity violations were corrected using the Greenhouse-Geisser procedure. Pairwise between-groups comparisons were performed and Bonferroni correction applied.

Regression Model

For all regression models, we additionally excluded the last trial of each block, as no learning rate could be estimated for these trials. All regression models were run at the participant level and reported statistics were calculated on group level averages.

In order to estimate how much participants updated their action according to the benchmark Bayesian model, we ran a linear regression model with four regressors: (a) absolute prediction error ($|\delta|$), (b) change-point probability (Ω), (c) model confidence (υ) and (d) hit/missed as categorical predictor. Model confidence was inserted as (1-CPP)*(1-MC) to reflect the second component of the learning rate in Equation 4. The second and third regressors should be positive (and close to 1) if participant behavior approximates the benchmark Bayesian model. Since both components were linearly predictive of learning rate in the benchmark Bayesian model, all regressors – except PE – were implemented as interaction terms with PE. The last regressor coded whether the particle was caught or could alternatively be seen as positive feedback. This information was unavailable to the benchmark Bayesian model and thus the model would not predict any influence of this term. The dependent variable 'action' was constructed similar to the update formula of the quasi-optimal model, by multiplying the LR $(\widehat{\alpha})$ by the absolute spatial prediction error $(\widehat{\alpha}_t \times |\widehat{\delta}_t|)$.

For reported confidence we ran a linear regression model with similar predictors, now without the interaction term with PE. All predictors were z-scored at the subject level. The dependent variable 'confidence' was the z-scored reported confidence provided by the participants. As confidence increases when uncertainty decreases, we expect negative beta weights for the parameters of CPP and model confidence.

Regression fits were as follows: action controls, median r^2 = 0.814; action patients, median r^2 = 0.846; confidence controls, median r^2 = 0.152; confidence patients, median r^2 = 0.110. The posterior model probability was used for Bayesian model selection (Stephan et al., 2009) among a finite set of models (Table S4; Figure S2). Wilcoxon rank-sum test was used for between-group comparisons on values of the predictors of the selected regression models (Figures 3C, 3F, and 4A).

Finally, to bring together the results of the previous two regression models, we wanted to investigate whether the link between confidence- and action-updating was in fact weakened in the patient group (Figure 4A). We therefore constructed a new regression model with the absolute confidence-update (i.e., the absolute difference between z-scored confidence reports on trial t and t-1) as the independent variable and absolute action-update (i.e., the absolute difference between where participants positioned bucket on trial t and t-1) as the dependent variable. We reasoned that if confidence and action were linked, then on trials where the participant had to adjust the position of the bucket more, confidence reports would also have to be adjusted more, irrespective of directionality (hence the absolute). Pearson's correlation was also used to measure the association between symptom severity and strength of coupling between action control and metacognitive report of confidence (Figure 4B).

DATA SOFTWARE AVAILABILITY

Data and Code Availability

The data and the analysis scripts are available on https://github.com/BDMLab and https://doi.org/10.17863/CAM.13236 for raw, unprocessed behavioral data.

Neuron, Volume *96*

Supplemental Information

Compulsivity Reveals a Novel Dissociation between Action and Confidence

Matilde M. Vaghi, Fabrice Luyckx, Akeem Sule, Naomi A. Fineberg, Trevor W. Robbins, and Benedetto De Martino

Supplementary Material

Table S1. Demographic and Clinical Characteristics of the Participants Included in the Study. Related to STAR Methods.

	Controls	OCD Patients		
Gender (M:F)	13:12	13:11	$\chi^2_1 = 0.023$	$p = 0.879$
Age	40.68 (10.19)	41.33 (12.32)	$t_{47} = -0.203$	$p = 0.840$
Verbal IQ	117.92 (3.97)	116.32 (2.99)	$t_{46} = 1.568$	$p = 0.124$
MADRS	1.36 (2.02)	9.92 (4.36)	$t_{47} = -8.870$	$p < 0.001$
OCI-R	7.64 (6.37)	29.25 (11.87)	$t_{47} = -7.984$	$p < 0.001$
STAI-State	28.90 (7.06)	43.50 (10.72)	$t_{47} = -5.496$	$p < 0.001$
STAI-Trait	35.00 (7.01)	56.58 (7.91)	$t_{47} = -10.120$	$p < 0.001$
Y-BOCS Total	-	22.75 (4.32)		
Y-BOCS Obsessions	-	10.79 (2.41)		
Y-BOCS Compulsions	-	11.96 (2.23)		

Mean values are reported and standard deviation in brackets.

Table S2. List of Quantiles for Error Magnitude and Values of Learning Rate for Each Quantile. Related to Figure 2B.

Quantile	Boundaries quantiles	CTL		OCD	
		Mean	(SEM)	Mean	(SEM)
**1**	[1-2)	0.000	(0.00)	0.088	(0.053)
**2**	[2-3)	0.010	(0.017)	0.281	(0.082)
**3**	[3-4)	0.038	(0.032)	0.357	(0.142)
**4**	[4-5)	0.052	(0.034)	0.334	(0.114)
**5**	[5-7)	0.075	(0.038)	0.342	(0.094)
**6**	[7-8)	0.110	(0.049)	0.376	(0.097)
**7**	[8-9)	0.106	(0.055)	0.334	(0.090)
**8**	[9-11)	0.112	(0.034)	0.352	(0.083)
**9**	[11-12)	0.121	(0.042)	0.366	(0.090)
**10**	[12-13)	0.154	(0.055)	0.370	(0.087)
**11**	[13-15)	0.184	(0.042)	0.420	(0.077)
**12**	[15-17)	0.225	(0.049)	0.455	(0.077)
**13**	[17-19)	0.288	(0.044)	0.506	(0.063)

**14**	[19-22)	0.402	(0.043)	0.588	(0.047)
**15**	[22-25)	0.484	(0.044)	0.689	(0.041)
**16**	[25-29)	0.521	(0.043)	0.731	(0.036)
**17**	[29-37)	0.632	(0.045)	0.826	(0.040)
**18**	[37-66)	0.764	(0.037)	0.917	(0.035)
**19**	[66-125)	0.917	(0.020)	0.929	(0.019)
**20**	[125-180)	0.883	(0.021)	0.927	(0.019)

For values presented in Figure 2B the distribution of the values of the spatial prediction error (i.e., $\hat{\delta}_t = X_t - b_t$, Equation 2) was divided in 20 quantiles. The table displays boundaries of each quantile, and associated mean (and SEM in brackets) of the learning rate ($\hat{\alpha}$) for controls and OCD patients.

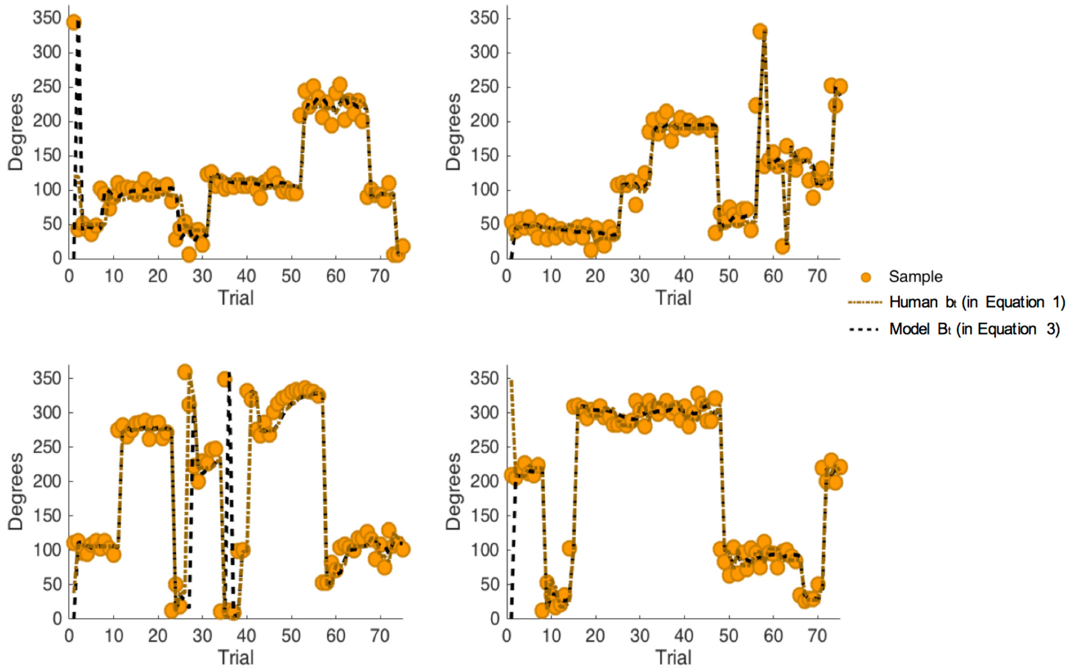

Figure S1. Comparison Between Human Participants and Model. Related to Figure 3B. Comparison was performed at the trial-by-trial level between subject's bucket position b_t computed in Equation 1 and model term B_t computed with Equation 3. Orange data points mark the location at which successive particles landed. The black dashed line marks the prediction of a quasi-optimal Bayesian model (i.e., B_t model belief update computed according to Equation 3, $B_{t+1} = B_t + \alpha_t \times \delta_t$). The brown dashed line marks the bucket's position (i.e., b_t in Equation 1 where participant positioned the bucket from one trial to the

next). Data are shown for one representative control subject. Each panel represents an experimental run of 75 trials.

Table S3. Summary of Parameters Obtained from the Regression Model for Action and Confidence[a]. Related to Figures 3C and 3F.

	Group	Beta	SEM	Within groups comparison			Between groups comparison		
					Stat	p	z	Rank sum	p
**Action**									
PE	CTL	0.264	0.077	t_{24}=	3.422	0.002	-2.370	506	0.018
	OCD	0.567	0.069	t_{23}=	8.197	<0.001			
CPP	CTL	0.565	0.056	t_{24}=	10.076	<0.001	2.530	752	0.011
	OCD	0.314	0.065	t_{23}=	4.856	<0.001			
MC	CTL	0.856	0.191	t_{24}=	4.479	<0.001	0	625	1
	OCD	0.632	0.130	t_{23}=	4.859	<0.001			
Hit/Missed	CTL	-0.742	0.053	t_{24}=	-13.978	<0.001	1.650	708	0.098
	OCD	-0.863	0.052	t_{23}=	-16.586	<0.001			
**Confidence**									
PE	CTL	-0.061	0.034	t_{24}=	-1.786	0.087	0.370	644	0.711
	OCD	-0.060	0.039	t_{23}=	-1.538	0.138			
CPP	CTL	-0.205	0.059	t_{24}=	-3.478	0.002	-1.170	566	0.242
	OCD	-0.107	0.049	t_{23}=	-2.157	0.042			
MC	CTL	-0.214	0.041	t_{24}=	-5.281	<0.001	-1.230	563	0.219
	OCD	-0.139	0.030	t_{23}=	-4.642	<0.001			
Hit/Missed	CTL	0.204	0.030	t_{24}=	6.854	<0.001	-0.330	608	0.741
	OCD	0.213	0.027	t_{23}=	7.832	<0.001			

[a] PE, prediction error; CPP, change-point probability; MC, model confidence.

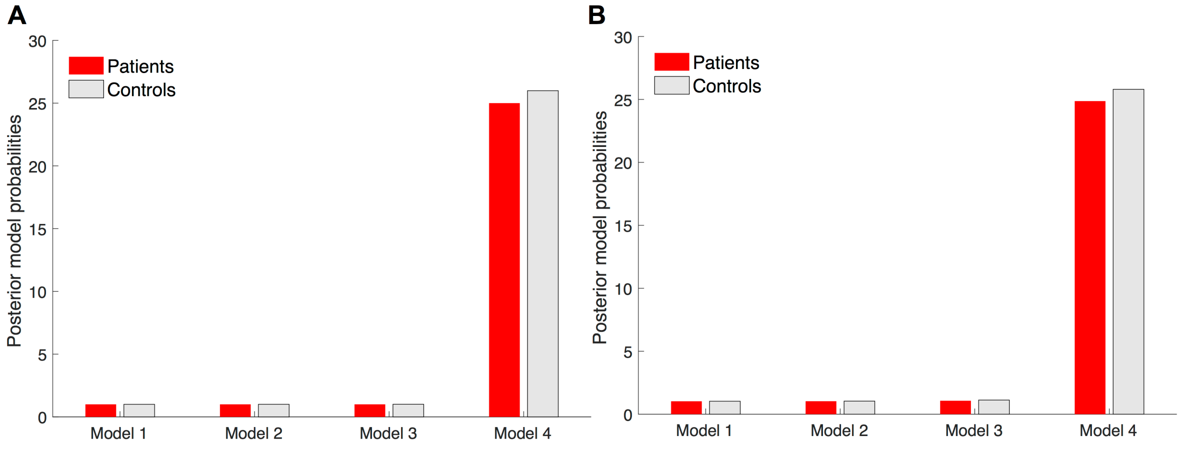

Figure S2. Bayesian Model Selection. Related to STAR Methods. (A) Posterior 'action regression' model probabilities after Bayesian model selection. The full and partial regression models predicting action were compared using the spm_BMS function (Stephan et al., 2009) from the SPM12 toolbox in Matlab (http://www.fil.ion.ucl.ac.uk/spm/software/spm12/). For both groups, the full regression model (Model 4, Table S4, Action) was deemed the most probable model. **(B)** Posterior 'confidence regression' model probabilities after Bayesian model selection. The best model to explain the data for both groups was again Model 4 (Table S4, Confidence), the full model with all four predictors included.

Table S4. Formulae for Models Represented in Figure S2 [a]. Related to STAR Methods.

Models	Formulae
**Action**	
**Model 1**	Action ~ $ß_0$ + $ß_1$[Prediction error] + ε
**Model 2**	Action ~ $ß_0$ + $ß_1$[Prediction error] + $ß_2$[CPP*PE] + ε
**Model 3**	Action ~ $ß_0$ + $ß_1$[Prediction error] + $ß_2$[CPP*PE] + $ß_3$[(1-CPP)*(1-MC)*PE] + ε
**Model 4**	Action ~ $ß_0$ + $ß_1$[Prediction error] + $ß_2$[CPP*PE] + $ß_3$[(1-CPP)*(1-MC)*PE] + $ß_4$[Hit*PE] + ε
**Confidence**	
**Model 1**	Confidence ~ $ß_0$ + $ß_1$[Prediction error] + ε
**Model 2**	Confidence ~ $ß_0$ + $ß_1$[Prediction error] + $ß_2$[CPP] + ε
**Model 3**	Confidence ~ $ß_0$ + $ß_1$[Prediction error] + $ß_2$[CPP] + $ß_3$[(1-CPP)*(1-MC)] + ε
**Model 4**	Confidence ~ $ß_0$ + $ß_1$[Prediction error] + $ß_2$[CPP] + $ß_3$[(1-CPP)*(1-MC)] + $ß_4$[Hit] + ε

[a]PE, prediction error; CPP, change-point probability; MC, model confidence.

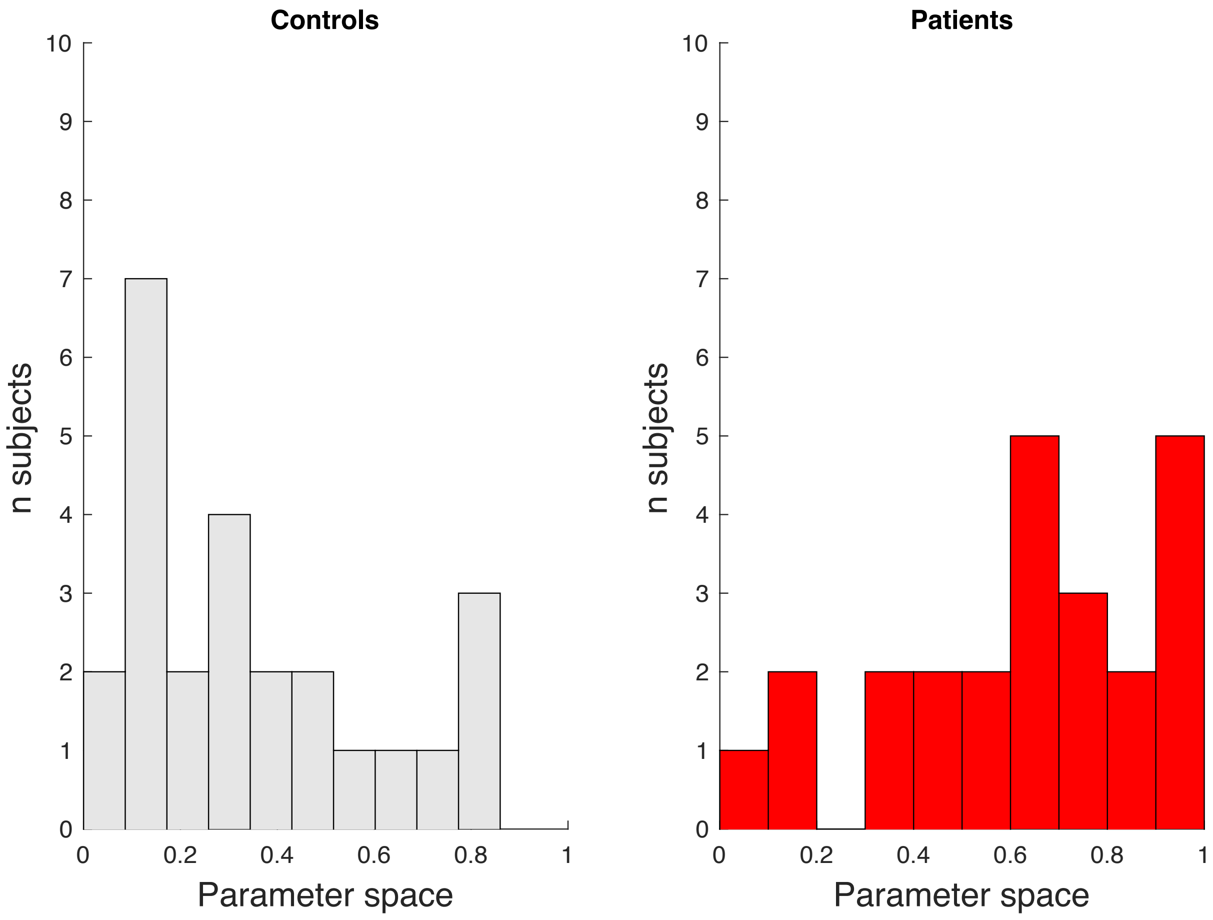

Figure S3. Distribution of Fitted Hazard Rates for Each Group. Related to STAR Methods. Model fits were obtained after an exhaustive search for possible values between 0 and 1. Best fitting parameters were determined by minimum least-squares fits between subject and model choices on each trial. The distribution of model fits showed a clear bimodal distribution, with overall higher hazard rate fits for patients (median = .62) compared to controls (median = .32, Wilcoxon rank-sum test, p = .007). However, these results are not surprising, as hazard rate is highly correlated with learning rate (see Nassar et al., 2012). Furthermore, we did not include these fits in our main analyses as those focused on subjects' behavior compared to a benchmark Bayesian model.

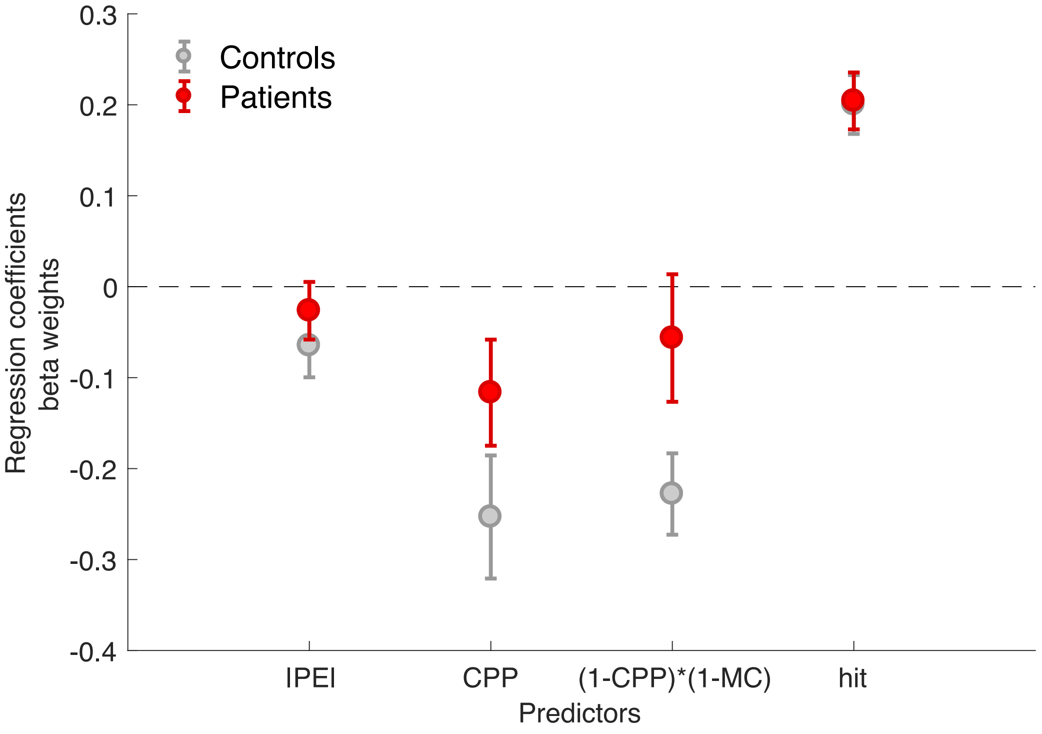

Figure S4. Regression on Confidence for Model with Fitted Hazard Rate. Related to STAR Methods. We ran an identical regression model as Figure 3F, with one change: model regressors CPP and (1-CPP)*(1-MC) were no longer derived from the benchmark Bayesian model, but from the model with fitted hazard rate for each participant. Results showed that model confidence no longer predicted patients' confidence (MC: OCD, -0.06±0.07, t_{19}=0.79, p=0.44), and this was significantly higher compared to controls (CTL, -0.23±0.04; paired two-sided t-test, p=0.04). Error bars represent SEM. Note that four participants from the patient group were excluded from the analysis, as their hazard rate was close to 1, in which case the two model predictors are perfectly negatively correlated and the regression model cannot find good fits. This analysis provides a different way of showing the dissociation of action and confidence in the OCD group: when hazard rate is fitted to participants' action (i.e. bucket update) OCD behavior is fitted by higher hazard rate, in other words OCD actions were reflecting an increased *perceived* volatility in the environment. However, OCD confidence rating did not reflect this increased *perceived* volatility (estimated using the fitted model) but were more aligned with the actual level of volatility in the environment. This analysis might provide another indication that patients did indeed understand the properties of the environment, but failed to act upon them.
